# Mapping of Imprinted Quantitative Trait Loci Using Immortalized F_2_ Populations

**DOI:** 10.1371/journal.pone.0092989

**Published:** 2014-03-27

**Authors:** Yongxian Wen, Weiren Wu

**Affiliations:** 1 Key Laboratory of Education Ministry for Genetics, Breeding and Multiple Utilization of Crops, Fujian Agriculture & Forestry University, Fuzhou, Fujian, China; 2 School of Computer and Information Science, Fujian Agriculture & Forestry University, Fuzhou, Fujian, China; The Australian National University, Australia

## Abstract

Mapping of imprinted quantitative trait loci (iQTLs) is helpful for understanding the effects of genomic imprinting on complex traits in animals and plants. At present, the experimental designs and corresponding statistical methods having been proposed for iQTL mapping are all based on temporary populations including F_2_ and BC_1_, which can be used only once and suffer some other shortcomings respectively. In this paper, we propose a framework for iQTL mapping, including methods of interval mapping (IM) and composite interval mapping (CIM) based on conventional low-density genetic maps and point mapping (PM) and composite point mapping (CPM) based on ultrahigh-density genetic maps, using an immortalized F_2_ (imF_2_) population generated by random crosses between recombinant inbred lines or doubled haploid lines. We demonstrate by simulations that imF_2_ populations are very desirable and the proposed statistical methods (especially CIM and CPM) are very powerful for iQTL mapping, with which the imprinting effects as well as the additive and dominance effects of iQTLs can be unbiasedly estimated.

## Introduction

Genomic imprinting is an epigenetic phenomenon in which some genes show non-equivalent allele expression depending on parental origins [1]. In terms of the parental origins of alleles, the heterozygotes at a locus with two different alleles can be divided into two reciprocal types. The differential allele expression of an imprinted gene may result in phenotypic difference between the reciprocal heterozygotes, according to which the imprinted gene can be identified. A large number of imprinted genes controlling various traits have been identified in human [2]–[6], animals [7], [8] and plants [9]–[12], implying that genomic imprinting occurs widely in animals (including human) and plants. Recently, due to the advent of high-throughput RNA sequencing technology, direct genome-wide survey of imprinted genes at the transcription level has become possible [13], [14], but the phenotypic effects of these putative imprinted genes remain to be investigated.

For complex traits, some quantitative trait loci (QTLs) may also exhibit imprinting effects (i.e., show different genotypic values between reciprocal heterozygotes) and hence are termed imprinted QTLs (iQTLs). Evidence has shown that imprinting effects are almost as prevalent as additive effects in some cases [15]. For example, ∼60% of the mapped QTLs underlying multiple metabolic traits in mouse such as adiposity, serum lipid levels and diabetes-related traits had imprinting effects [15]. Therefore, identification of iQTLs is important for the full understanding of phenotypic variation in complex traits.

To identify an iQTL based on its imprinting effect, it is necessary to distinguish the reciprocal heterozygotes or the parental origins of alleles at the iQTL. For this purpose, appropriate experimental designs and corresponding statistical methods are required. The F_2_ generation of a cross between two either inbred or outbred lines is suitable for analyzing various QTL effects (including imprinting effects) because it contains all possible genotypes at a locus with two different alleles (including two different homozygotes and two reciprocal heterozygotes). The outbred F_2_ design is most convenient for outbred species. In this design, the origins of alleles at informative marker loci (possessing more than two alleles) in the F_2_ generation can be traced back to the F_1_ parents and the founder grandparents [16]. Therefore, it is suitable for genome-wide mapping of iQTLs [7], [17], [18]. The inbred F_2_ design is convenient for inbred species and also applicable to outbred species. However, the parental origins of marker alleles in the inbred F_2_ generation cannot be directly determined because the F_1_ parents are identical genetically. Nevertheless, based on the variation of recombination rate between different sexes, the parental origins of haplotypes can be distinguished [19] and therefore iQTL mapping can still be performed [20]–[22]. The BC_1_ generation of inbred line cross has also been proposed for iQTL mapping, in which the parental origins of marker alleles can be inferred directly [21], [23], [24].

Although F_2_ and BC_1_ generations can be used for iQTL mapping, they all suffer some problems. In the outbred F_2_ design, only some genomic regions are informative for inferring the parental origins of alleles [15], [25] and the assumption that the founder lines are fixed for QTL differences but have segregating marker variation may be violated so that the imprinting effects detected may be false [15], [20]. The inbred F_2_ design is appropriate only for the species with large sex difference in recombination rate and lacks power when the difference is small due to high error rate [15]. In the BC_1_ design, imprinting effects and maternal genetic effects are fully confound [15]. In addition, F_2_ and BC_1_ generations are both temporary populations, which can be used only once.

**Figure 1:**
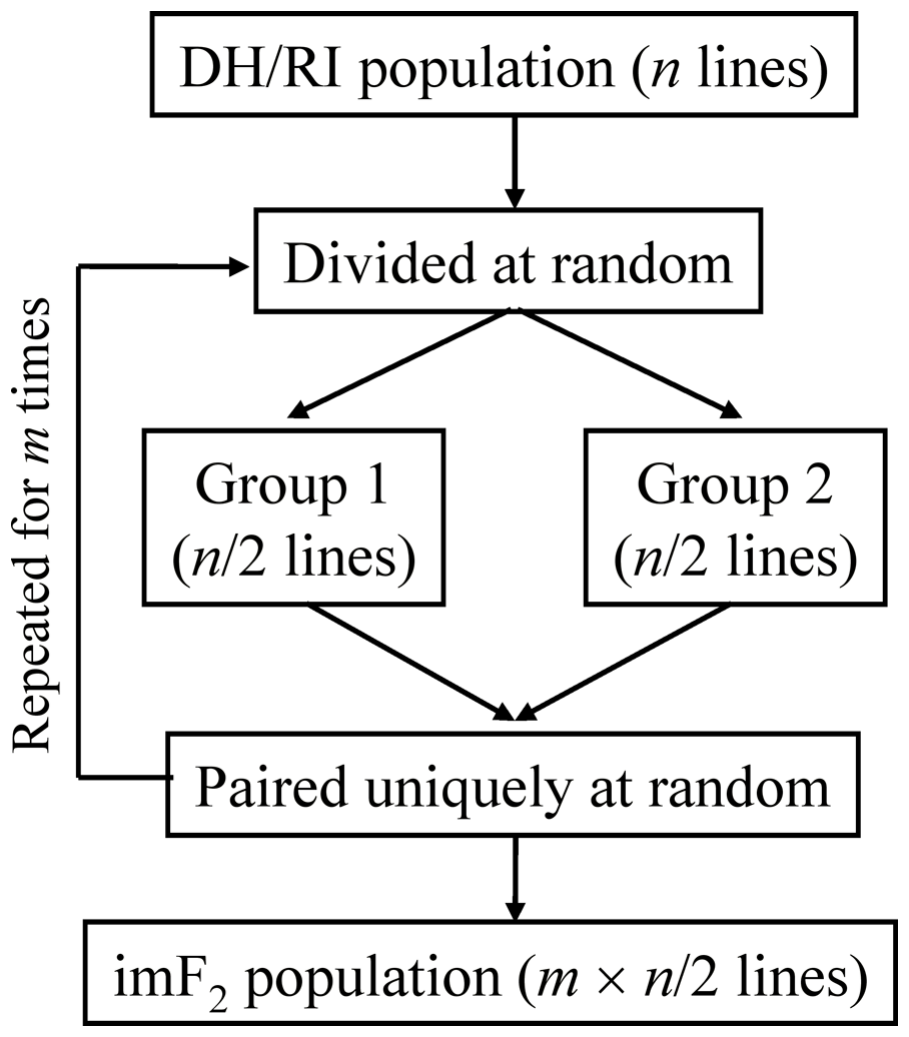
Diagram of the procedure for constructing an immortalized F_2_ population by randomly crossing DH/RI lines in a balanced way.

Random crosses between recombinant inbred (RI) lines or doubled haploid (DH) lines can result in a population of hybrid lines, of which the genetic structure is analogous to that of an F_2_ population (Fig. 1). As RI and DH populations are permanent populations, the hybrid line population can be produced repeatedly. Hence, it is called immortalized F_2_ (abbreviated as imF_2_) population [26] or recombinant inbred intercross (RIX) population in the case of using an RI population as the founders [27]. Because an imF_2_ population combines the merits of an F_2_ population and a permanent population, it is a very useful experimental design for genetic studies, which has been used in some important crop species such as rice [26], maize [28], wheat [29] and oilseed rape [30] and the model mammal mouse [31].

An obvious merit of imF_2_ populations is that the origins of marker alleles in an imF_2_ line can be directly inferred from its parental RI or DH lines [26], [27]. Hence, an imF_2_ population can be used for iQTL mapping. In this paper, we propose a framework for iQTL mapping using an imF_2_ population. We demonstrate that the proposed methods are powerful for iQTL mapping and can obtain unbiased estimates of the imprinting effect as well as the additive and dominance effects of an iQTL.

## Materials and Methods

### Genetic model

Consider a QTL with two alleles, Q_1_ and Q_2_, in a diploid species. The two alleles can be combined into four genotypes: Q_1_Q_1_, Q_1_Q_2_, Q_2_Q_1_ and Q_2_Q_2_, with one allele (the former) from a male gamete and the other (the latter) from a female gamete in each genotype. Let *g*_11_, *g*_12_, *g*_21_ and *g*_22_ represent the genotypic values of the four genotypes (with *g*_11_≥*g*_22_). The additive effect (*a*), dominance effect (*d*) and imprinting effect (*i*) of the QTL are defined as: 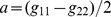, 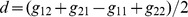, and 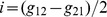 [32]. According to these definitions, a single-QTL model for imF_2_ population, in which the four QTL genotypes are segregated with equal proportions (i.e., 1/4 each), can be written as:
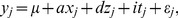
(1)where *y_j_* is the trait value of the *j*th imF_2_ line (*j* = 1, 2, …, *n*); *μ* is population mean; *ε_j_* is residual error following a normal distribution 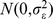; and *x_j_*, *z_j_* and *t_j_* are dummy variables taking values depending on the QTL genotype (Table 1).

**Table 1:** Values of dummy variables in Eq. (1) depending on the QTL genotype.

QTL genotype	*x_j_*	*z_j_*	*t_j_*
Q_1_Q_1_	1	0	0
Q_1_Q_2_	0	1	1
Q_2_Q_1_	0	1	−1
Q_2_Q_2_	−1	0	0

### Interval mapping of iQTLs

The values of the dummy variables in Eq. (1) are unknown because the QTL genotype is undetermined. To use Eq. (1) for iQTL mapping, it is necessary to know the probabilities of the four possible iQTL genotypes in an imF_2_ line. Since an imF_2_ line is the F_1_ progeny of two DH (or RI) lines, the probability of a QTL genotype (e.g. Q_1_Q_2_) in an imF_2_ line would be equal to the product of the probabilities of corresponding QTL genotypes in its paternal (e.g. Q_1_Q_1_) and maternal (e.g. Q_2_Q_2_) DH (or RI) lines. The probabilities of iQTL genotypes in a DH (or RI) line can be estimated in light of the genotypes of the flanking markers (Table 2). Thus, the probabilities of all possible iQTL genotypes in an imF_2_ line can be obtained (Table 3).

**Table 2:** Probabilities of QTL genotypes conditional upon the genotype of flanking markers in a DH (or RI) population.

Marker genotype	Symbol	No interference		Complete interference	
		Q_1_Q_1_	Q_2_Q_2_	Q_1_Q_1_	Q_2_Q_2_
A_1_A_1_B_1_B_1_	G_1_	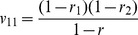	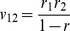	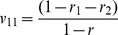	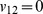
A_1_A_1_B_2_B_2_	G_2_	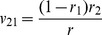	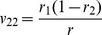	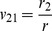	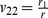
A_2_A_2_B_1_B_1_	G_3_	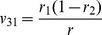	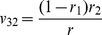	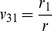	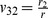
A_2_A_2_B_2_B_2_	G_4_	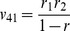	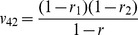	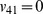	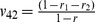

Note: *r*_1_, *r*_2_ and *r* are the recombination fractions between left marker A and QTL, between QTL and right marker B and between the two flanking markers. For RI population, *r* is replaced by an adjusted recombination fraction: *R* = 2*r*/(1+2*r*) for selfing and *R* = 4*r*/(1+6*r*) for brother-sister mating (similarly for *r*_1_ and *r*_2_).

**Table 3:** Probabilities of various QTL genotypes in an imF_2_ line conditional upon the cross combination between DH (or RI) lines.

Cross combination	Q_1_Q_1_	Q_1_Q_2_	Q_2_Q_1_	Q_2_Q_2_
G*_k_*×G*_l_*	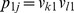	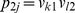	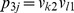	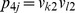

Note: See Table 2 for the meanings of G*_k_*, G*_l_*, *v_k_*_1_, *v_k_*_2_, *v_l_*_1_ and *v_l_*_2_ (*k*, *l* = 1, 2, 3, 4). Subscript *j* indicants the *j* ^th^ imF2 line (*j* = 1, 2, …, *n*).

According to Tables 1, 2 and 3, the expected values of the dummy variables in Eq. (1) can be obtained: 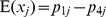, 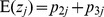, and 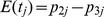. Let the dummy variables take their expected values. Then, Eq. (1) becomes a linear regression model, with which simplified interval mapping (IM) methods based on least squares estimation can be formulated [33]. To map iQTLs, we can scan the genome by examining imprinting effect displayed at every position using the following approximate log-likelihood ratio test:
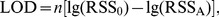
(2)where RSS_0_ and RSS_A_ are the minimum residual sum of squares of Eq. (1) under null hypothesis *H*_0_: *i* = 0 and alternative hypothesis *H*_A_: *i*≠0, respectively. The LOD significance threshold can be estimated via permutation tests [34]. A genomic region covered by a LOD peak exceeding the threshold is thought to contain an iQTL and the highest point of the peak is the most probable position of the iQTL.

### Composite interval mapping of iQTLs

Based on the IM method described above, the method of composite interval mapping (CIM) [35] can be further formulated by incorporating some background markers that display significant phenotypic effects as cofactors into Eq. (1). The purpose of using cofactors is to control genetic background noise caused by other QTLs than the putative one being tested. As phenotypic effect can be resolved into three orthogonal components (i.e., additive effect, dominance effect and imprinting effect), cofactors can be divided into three independent types, namely, the additive effect cofactor (AEC), dominance effect cofactor (DEC) and imprinting effect cofactor (IEC). The three types of cofactors are selected independently. The selection can be carried out by stepwise regression. For a marker selected, it is not necessary that all the three effect components are selected as cofactors, but only the significant ones are selected. This means that the three types of cofactors may correspond to different sets of markers. Thus, the model used for CIM in an imF_2_ population can be written as
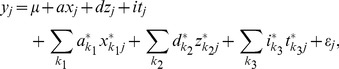
(3)where 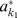, 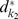 and 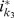 are the effects of the *k*_1_th AEC, *k*_2_th DEC and *k*_3_th IEC, respectively; 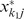, 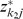 and 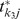 are dummy variables taking values depending on the genotypes of the corresponding markers in the *j*th imF_2_ line following the same rule for QTL (Table 1); and ∑ indicates summation over the cofactors; all the other symbols have the same meanings as those in Eq. (1). Similarly, the model of Eq. (3) can be fitted using least squares by letting the dummy variables *x*, *z* and *t* take their expected values, and the imprinting effect of the putative iQTL can be tested using formula (2), where RSS_0_ and RSS_A_ represent the minimum residual sum of squares of Eq. (3) under the null and alternative hypotheses, respectively. The LOD significance threshold can also be estimated via permutation tests [34]. In addition, to avoid statistical power reduction due to closely linked cofactors, a window is needed on each side of the target marker interval being tested. All the cofactors within the windows will be removed from the model.

### Mapping iQTLs based on ultrahigh-density genetic map

In recent years, the fast development of high-throughput next-generation sequencing (NGS) technologies has made it practical to obtain a huge number of single nucleotide polymorphism (SNP) markers for population genotyping by DNA sequencing directly [36]. This enables construction of ultrahigh-density genetic maps. For example, two ultrahigh-density genetic maps have been constructed based on RI populations in rice [37], [38]. In such maps, markers can well represent every position of the genome. Thus, QTL mapping can be performed by testing every marker directly without the need of scanning marker intervals. The model of Eq. (1) can be used for the marker test. But here, the values of the dummy variables *x*, *z* and *t* are determined. Therefore, least squares method can be used to fit the model, and similarly formula (2) can be used to test the imprinting effect of the marker (the putative iQTL). Again, the LOD significance threshold can also be estimated via permutation tests [34]. For distinction, we call this method as point mapping (PM). In addition, analogous to the extension from IM to CIM, PM can also be extended to composite point mapping (CPM) by adding cofactors into the model. The model fitting and testing in CPM is similar to that in CIM.

### Simulation studies

To examine the experimental design and statistical methods for iQTL mapping proposed above, we carried out three simulation studies. The first two studies simulated interval mapping of a single iQTL based on a conventional low-density genetic map. This was to examine the feasibility of using imF_2_ populations for iQTL mapping and investigate the factors that may influence the statistical power of iQTL mapping. The third study simulated genome-wide iQTL mapping using different statistical methods based on either a conventional low-density genetic map or an ultrahigh-density genetic map.

## Results

### Simulation study I

In this simulation study, we assumed that 1) the imF_2_ population used contained 500 lines generated from a DH population consisting of 200 lines; 2) an iQTL was located at the position of 55 cM on a chromosome, which was 100 cM in length and covered by 11 evenly-spaced markers; and 3) the imprinting effect of the iQTL explained 15% of the phenotypic variance in the imF_2_ population. Besides, five possible imprinting types [32] were considered (Tables 4 and 5). With the iQTL effects (*a*, *d* and *i*) and the heritability of imprinting effect (the proportion of phenotypic variance explained by the imprinting effect, denoted as 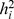) given, the residual variance (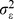) was determined by the following formula:
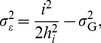
(4)where 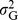 is the genetic variance of the iQTL:
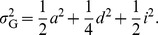
For each case, the simulation was replicated for 100 times, and a LOD threshold at the overall significance level of 0.05 was estimated by simulation (5000 replicates). The procedure of producing imF_2_ populations was as described in Fig. 1. The simulated data were analyzed using the IM method.

**Table 4:** Imprinting types and their definitions.

Imprinting type	Abbreviation	Definition
Parental expression, Paternal	PEP	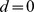 ∩ 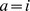
Parental expression, Maternal	PEM	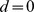 ∩ 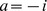
Dominance imprinting, Bipolar	DIB	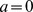 ∩ 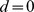
Dominance imprinting, Polar, Over-dominance	DIPOD	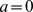 ∩ 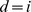
Dominance imprinting, Polar, Under dominance	DIPUD	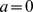 ∩ 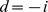

**Table 5:** Simulation results of mapping iQTLs of different imprinting types.

Type	Expected			Estimated (mean ± s.d.)			
	*a*	*d*	*i*	Position	*a*	*d*	*i*
PEP	2	0	2	54.75±3.58	1.98±0.36	0.05±0.35	1.96±0.27
PEM	2	0	−2	55.55±4.24	1.96±0.26	−0.03±0.30	−1.96±0.29
DIB	0	0	2	54.28±3.15	−0.03±0.17	−0.00±0.21	2.03±0.20
DIPOD	0	2	2	54.00±3.73	−0.02±0.20	2.01±0.38	1.99±0.27
DIPUD	0	2	−2	54.71±3.90	0.02±0.17	2.01±0.33	−2.02±0.25

Note: 100 replicates of simulation were performed for each type. The iQTL was assumed to be at the position of 55 cM. The statistical power of iQTL detection was 100% in all the types.

The results showed that both the position and the various effects of the iQTL were unbiasedly estimated in all the cases (Table 5), demonstrating that iQTL mapping based on imF_2_ populations is feasible.

### Simulation study II

In this simulation study, we investigated the influences of three factors, including the heritability of imprinting effect, the size of parental (DH or RI) population and the size of imF_2_ population, on the statistical power and accuracy of iQTL mapping. As these factors are not related to imprinting types, we only simulated the type “dominance imprinting, bipolar”. Namely, we set the iQTL effects as *a* = 0, *d* = 0, and *i* = 2. Three levels of the heritability of imprint effect (2%, 5% and 10%), two sizes of the parental DH population (100 and 200), and four sizes of the imF_2_ population (200, 300, 400, 500) were investigated (for the case of heritability = 2%, the four sizes of imF_2_ population were set as 200, 500, 800 and 1000). Again, for each case, the residual variance was determined by formula (4), the simulation was replicated for 100 times, and a LOD threshold at the overall significance level of 0.05 was estimated by simulation (5000 replicates).

The results indicated that the statistical power of iQTL detection and the precision of iQTL position and effect estimation are mainly influenced by the heritability of imprinting effect and the size of the imF_2_ population, but hardly influenced by the size of the parental DH population (Table 6). It is obvious that the power and precision raise as the increase of the heritability of imprinting effect and the imF_2_ population size. A population size of 200 imF_2_ lines appears to be large enough for efficient detection (power >95%) and precise mapping and effect estimation of an iQTL with medium heritability (10%), and so do a size of 400 for small (5%) heritability and that of 1000 for very small (2%) heritability, respectively.

**Table 6:** Simulation results of iQTL mapping under different heritabilities and different population sizes.

Heritability (%)	Population size		QTL position (cM)	QTL effects			Power (%)
	DH	imF_2_		*a*	*d*	*i*	
2	100	200	53.33±17.04	0.05±1.06	−0.08±2.03	3.42±0.53	30
		500	54.94±13.81	0.00±0.73	−0.12±1.01	2.37±0.46	78
		800	55.44±9.02	−0.00±0.54	−0.03±0.77	2.15±0.381	87
		1000	53.35±8.05	−0.08±0.44	0.07±0.74	2.07±0.39	96
	200	200	55.91±13.16	0.03±1.02	0.31±1.51	3.45±0.56	34
		500	54.59±14.55	−0.02±0.62	0.00±1.02	2.48±0.44	71
		800	53.65±8.95	−0.09±0.53	−0.02±0.77	2.23±0.39	94
		1000	53.41±7.38	−0.01±0.47	−0.03±0.65	2.09±0.43	96
5	100	200	55.64±9.59	−0.08±0.66	0.12±0.99	2.40±0.44	76
		300	55.95±9.89	0.00±0.49	−0.03±0.85	2.12±0.38	86
		400	54.76±7.97	−0.01±0.51	0.17±0.68	2.05±0.42	96
		500	54.52±9.24	−0.05±0.40	0.10±0.65	2.04±0.38	98
	200	200	55.40±17.66	0.02±0.84	−0.01±1.19	2.34±0.77	77
		300	54.51±10.35	0.03±0.48	0.02±0.92	2.03±0.55	84
		400	55.61±7.40	0.00±0.46	0.05±0.73	2.08±0.46	97
		500	55.01±7.26	−0.03±0.41	0.03±0.64	2.01±0.40	100
10	100	200	54.67±10.86	−0.02±0.48	−0.05±0.64	2.07±0.39	98
		300	55.22±8.31	0.03±0.31	0.04±0.56	2.01±0.36	100
		400	54.43±3.68	0.04±0.36	0.03±0.46	1.97±0.34	100
		500	55.10±4.62	−0.01±0.29	0.04±0.42	2.03±0.30	100
	200	200	56.97±8.52	0.06±0.47	0.01±0.69	2.05±0.42	98
		300	55.23±7.40	−0.02±0.42	0.00±0.53	2.03±0.39	100
		400	54.34±5.05	−0.06±0.32	−0.02±0.44	1.99±0.32	100
		500	54.68±3.46	0.05±0.31	0.02±0.47	2.04±0.27	100
Real value			55	0	0	2	

### Simulation study III

In this simulation study, we considered an example of iQTL mapping in a whole genome. We assumed that a diploid species had 3 pairs of chromosomes, each of which was 150 cM long. There were 3, 1 and 2 iQTLs on chromosomes 1, 2 and 3, respectively, and also 1 non-imprinted QTL (QTL4) on chromosome 2 (Table 7). An imF_2_ population of 1000 hybrid lines was generated from a DH population of 200 lines. The population mean and the environmental variance were set to be 10 and 6, respectively. Based on simulated samples, the phenotypic variance of the imF_2_ population was estimated to be 12.4. Therefore, the broad sense heritability of the trait was estimated to be 51.6%, and the heritabilities of imprinting effect of individual iQTLs were estimated to vary between 1.65% and 8.36%; the non-imprinting QTL had null heritability of imprinting effect (Table 7). In regard to the genetic map used for iQTL mapping, two cases (examples) were simulated. In the first example, a conventional low-density map was assumed, in which 16 markers were evenly distributed on each chromosome, with a space of 10 cM between adjacent markers. In the second example, an ultrahigh-density map was assumed, in which there was one marker every 1 cM. The data of Example I were analyzed with the methods of IM and CIM, while those of Example II were analyzed with the methods of PM and CPM. Cofactors for CIM and CPM were selected by stepwise regression at the significance level of 0.05. A 10 cM window and a 5 cM window were used in CIM and CPM, respectively. LOD significance thresholds at the overall significance level of 0.05 were estimated by permutation tests (1000 replicates).

**Table 7:** Simulation results of genome-wide iQTL mapping based on a low-density genetic map (using IM and CIM methods) and an ultrahigh-density genetic map (using PM and CPM methods), respectively.

		chromosome 1			chromosome 2		chromosome 3	
		QTL1	QTL2	QTL3	QTL4	QTL5	QTL6	QTL7
Real value	Position (cM)	17	78	133	67	85	81	105
	*a*	1.1	0	−1.2	1.04	0	0	0
	*d*	0	0.8	0	2	0.9	0	0.98
	*i*	1.1	−0.8	1.2	0	0.9	−1.08	0.98
	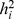 (%)	5.9	1.65	8.36	0	2.65	5.49	3.72
	Imprinting type	PEP	DIPUD	PEM	Non	DIPOD	DIB	DIPOD
Estimate								
IM	Position (cM)	20		128		86		90
	*a*	1.15		−0.86		0.70		0.17
	*d*	0.02		−0.02		2.06		0.31
	*i*	0.62		1.07		0.78		−0.66
CIM	Position (cM)	20	82	132		90	80	106
	*a*	0.92	−0.22	−1.05		−0.13	−0.01	0.55
	*d*	−0.23	0.71	−0.04		1.03	0.40	0.38
	*i*	0.71	−0.54	1.20		0.83	−0.87	1.47
PM	Position (cM)	16		130		82		106
	*a*	0.84		−1.00		0.66		0.39
	*d*	0.34		−0.05		1.90		1.13
	*i*	0.80		1.23		0.89		0.63
CPM	Position (cM)	16	77	132		81	80	104
	*a*	1.10	−0.07	−1.51		0.32	0.05	0.11
	*d*	0.24	0.85	0.11		0.63	−0.02	1.05
	*i*	1.00	−0.86	1.66		0.86	−1.52	1.20

**Figure 2:**
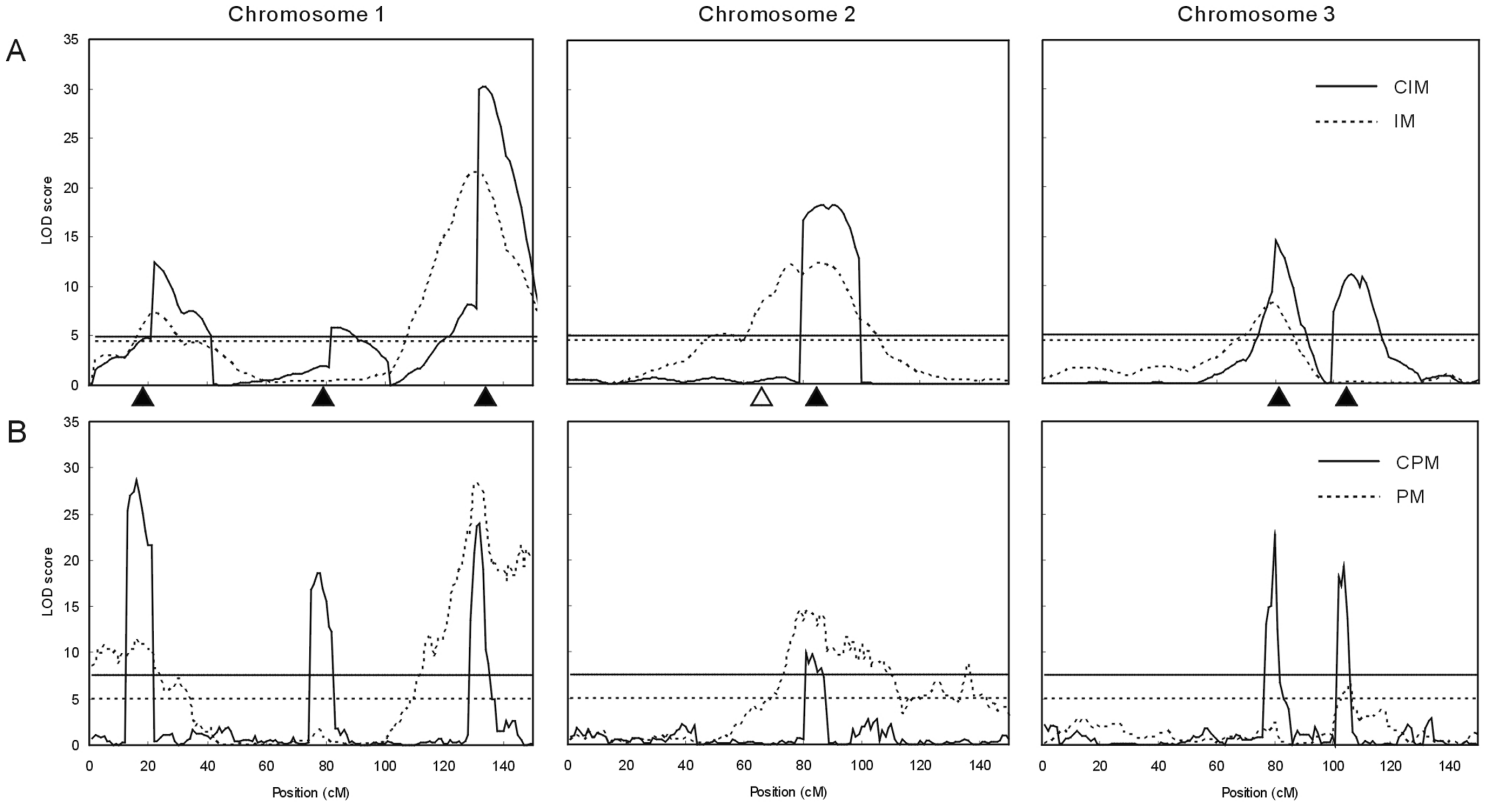
Simulation results of genome-wide iQTL mapping using a conventional low-density genetic map (A) and an ultrahigh-density map (B), respectively. The horizontal lines indicate the significance threshold at the overall significance level of 0.05. The black and white triangles indicate the positions of iQTLs and non-imprinted QTLs, respectively.

The results are shown in Table 7 and Fig. 2. As expected, the non-imprinted QTL (QTL4) could not be detected in all the cases. CIM and CPM could detect all the 6 iQTLs, whereas IM and PM could only detect four of them. Besides, in Example II, PM appeared to detect a false iQTL on chromosome 2 (Fig. 2B). These results indicate that CIM and CPM are more powerful than IM and PM, respectively, demonstrating the benefit of incorporating cofactors in the model. By comparing the results of CIM and CPM, it is seen that the LOD profile peaks obtained by CPM are much sharper and narrower than those obtained by CIM (Fig. 2), suggesting that high marker density can increase the resolution of iQTL mapping.

## Discussion

We have proposed a framework for iQTL mapping using imF_2_ populations. The simulation studies demonstrate that an iQTL can be precisely mapped and its imprinting effect as well as additive and dominance effects can be unbiasedly estimated by the simple IM method when only one iQTL is involved (Tables 5 and 6); in the case of genome-wide iQTL mapping, both CIM and CPM can achieve satisfactory statistical power and mapping precision (Table 7; Fig. 2). These results indicate that imF_2_ populations are quite suitable and the proposed statistical methods are very powerful for iQTL mapping.

All the three types of cofactors (AEC, DEC and IEC) used in CIM and CPM are helpful for iQTL mapping, but their roles may be different. Because only imprinting effect is tested in iQTL mapping, it is expectable that IECs must be the most important. Indeed, we have found by simulation that the LOD profile obtained by CIM (or CPM) is similar in shape to (though generally higher in value than) that obtained by IM (or PM) when only AECs and DECs (but no IECs) are included in the regression model (data not shown). This result suggests that whilst IECs can affect both statistical power and mapping precision, AECs and DECs mainly influence statistical power but have little impact on mapping precision.

Determination of the parental origins of marker alleles is a prerequisite for iQTL mapping. An imF_2_ population is generated from random crosses between RI or DH lines. In theory, the genetic segregation at a locus in an RI or DH population is analogous to that among the gametes generated by a heterozygote. Hence, the construction of an imF_2_ population is genetically equivalent to an artificially controlled process of random combination between male and female gametes. As the marker genotypes in RI or DH lines are known, the parental origins of marker alleles in imF_2_ lines can be exactly determined by genetic inference. This is a particular and significant merit of the imF_2_ design for iQTL mapping compared with the outbred F_2_ and inbred F_2_ designs, where the parental origins of marker alleles or haplotypes are inferred based on probabilities [18], [20], [22], which may reduce the power of iQTL mapping due to the uncertainty.

In addition, as the hybrid of two pure lines, an imF_2_ line is a genetically homogeneous line. Hence, similar to RI and DH populations, imF_2_ populations allow replicated trials and measurements on the same genotypes. This can effectively reduce environmental variation so as to increase the power of iQTL mapping, and also enables the analysis of iQTL-by-environment interactions. Besides, as mentioned above, the marker genotype in an imF_2_ line can be deduced from its parental RI or DH lines. Therefore, no additional cost is needed on molecular marker assay in the construction of an imF_2_ population. Furthermore, an RI or DH population of medium size can form a great number of cross combinations. For example, 100 RI or DH lines can form 4950 cross combinations. Therefore, very large imF_2_ populations can be developed, which can greatly increase the power of iQTL mapping, as demonstrated in our simulation studies (Table 6). This is especially desirable when an ultrahigh-density genetic map is available, which provides a potential to achieve a very high precision of iQTL mapping as shown in our simulation results (Fig. 2), depending on the size of the imF_2_ population (which determines the statistical power) and also that of the parental DH or RI population (which determines the degree of recombination in the genome).

In summary, imF_2_ populations are an ideal experimental design possessing many desirable features for iQTL mapping.
